# Oral findings of patients in the intensive care unit: a systematic review

**DOI:** 10.15649/cuidarte.3959

**Published:** 2025-02-27

**Authors:** María Paula Aranda Gómez, Elizabeth Chaparro Torres, Nicole Dayanna Hernández Abahunza, Anghela Catalina Vargas Carreño, Laura Viviana Herrera Sandoval, Yeny Zulay Castellanos Domínguez

**Affiliations:** 1 Universidad Santo Tomás, Bucaramanga, Colombia. mariapaula.aranda@ustabuca.edu.co Universidad Santo Tomás Universidad Santo Tomás Bucaramanga Colombia mariapaula.aranda@ustabuca.edu.co; 2 Universidad Santo Tomás, Bucaramanga, Colombia. elizabeth.chaparro@ustabuca.edu.co Universidad Santo Tomás Universidad Santo Tomás Bucaramanga Colombia elizabeth.chaparro@ustabuca.edu.co; 3 Universidad Santo Tomás, Bucaramanga, Colombia. nicoledayanna.hernandez@ustabuca.edu.co Universidad Santo Tomás Universidad Santo Tomás Bucaramanga Colombia nicoledayanna.hernandez@ustabuca.edu.co; 4 Universidad Santo Tomás, Bucaramanga, Colombia. anghelacatalina.vargas@ustabuca.edu.co Universidad Santo Tomás Universidad Santo Tomás Bucaramanga Colombia anghelacatalina.vargas@ustabuca.edu.co; 5 Universidad Santo Tomás, Bucaramanga, Colombia. laura.herrera01@ustabuca.edu.co Universidad Santo Tomás Universidad Santo Tomás Bucaramanga Colombia laura.herrera01@ustabuca.edu.co; 6 Universidad Santo Tomás, Bucaramanga, Colombia. yeny.castellanos@ustabuca.edu.co Universidad Santo Tomás Universidad Santo Tomás Bucaramanga Colombia yeny.castellanos@ustabuca.edu.co

**Keywords:** Oral Health, Intensive Care Units, Critical Care, Patients, Salud Bucal, Unidades de Cuidados Intensivos, Cuidados Críticos, Pacientes, Saúde Bucal, Unidades de Terapia Intensiva, Cuidados Críticos, Pacientes

## Abstract

**Introduction:**

Patients in intensive care units often experience a decline in oral health. Systematic reports of oral findings in these patients are scarce.

**Objective:**

To document oral cavity lesions in patients in Intensive Care Units.

**Materials and Methods:**

A systematic review was conducted, with a literature search across five databases, focusing on publications from 2018 to 2023. The identified articles were imported into the Mendeley reference manager; titles and abstracts were reviewed in pairs and under blinded conditions. Pre-selected articles were exported to the Rayyan application for eligibility assessment. Quality of the studies was assessed using the Joanna Briggs Institute (JBI) tool. This research is classified as risk-free and complies with copyright regulations by exercising citation rights (Law 1915 of 2018 and Law 1032 of 2006).

**Results:**

A total of 1553 articles were identified. Using the PRISMA methodology, 11 studies were included, with 54% retrieved from PubMed and 91% published in English. Toothbrushing was the most frequently documented oral care procedure. Gingivitis and ulcers were the most frequent oral findings.

**Discussion:**

The literature confirms the presence of oral findings in these patients, which are related to their systemic health status, procedures, equipment, and oral health care protocols.

**Conclusion:**

Although oral care is provided at a high rate in critically ill patients, oral alterations and lesions are frequently observed. The involvement of a dentist is essential for the comprehensive care of these patients.

## Resumen

**Introducción:**

Los pacientes en cuidados intensivos presentan deterioro en la salud bucal. Son escasos los reportes sistemáticos de los hallazgos bucales en estos pacientes.

**Objetivo:**

Documentar las lesiones presentes en cavidad bucal de pacientes que han permanecido en Cuidados Intensivos.

**Materiales y métodos:**

Revisión sistemática con búsqueda de literatura en cinco bases de datos, publicados entre 2018 y 2023. Se descargaron los artículos identificados en el gestor de referencias Mendeley; de forma pareada y cegada se revisó título y resumen. Para la verificación de elegibilidad los artículos preseleccionados se exportaron a la aplicación Rayyan. La evaluación de calidad se hizo con el instrumento Joanna Briggs Institute (JBI). Esta investigación es catalogada sin riesgo, en la que se respetaron derechos de autor ejerciendo el derecho de cita (Ley 1915 de 2018 y la 1032 de 2006).

**Resultados:**

Se identificaron 1553 artículos, mediante metodología PRISMA, se incluyeron 11 estudios, de éstos 54% recuperados de Pubmed, 91% publicados en inglés. El cuidado bucal más documentado fue el cepillado dental. De los hallazgos bucales la gingivitis y las úlceras fueron los eventos más frecuentes.

**Discusión:**

La literatura confirma la presencia de hallazgos bucales en estos pacientes relacionados con su estado de salud sistémico, procedimientos y equipamientos, así como protocolos y cuidados de salud bucal.

**Conclusiones:**

Aunque los cuidados bucales se brindan en alta proporción en pacientes en estado crítico, es frecuente la presencia de alteraciones y lesiones bucales. Es fundamental la participación del odontólogo en el cuidado integral de estos pacientes.

## Resumo

**Introdução:**

Pacientes em unidades de terapia intensiva frequentemente apresentam deterioração da saúde bucal Relatos sistemáticos de achados orais nesses pacientes são escassos.

**Objetivo:**

Documentar as lesões presentes na cavidade oral de pacientes que permaneceram em Terapia Intensiva.

**Materiais e métodos:**

Revisão sistemática com busca bibliográfica em cinco bases de dados, publicadas entre 2018 e 2023. Os artigos identificados foram baixados no gerenciador de referências Mendeley; de forma pareada e cega, o título e o resumo foram revisados. Para verificação de elegibilidade, os artigos selecionados foram exportados para o aplicativo Rayyan. A avaliação da qualidade foi feita com o instrumento Joanna Briggs Institute (JBI). Esta pesquisa está catalogada sem risco, na qual os direitos autorais foram respeitados através do exercício do direito de citação (Lei 1.915 de 2018 e 1.032 de 2006).

**Resultados:**

Foram identificados 1.553 artigos, utilizando a metodologia PRISMA, foram incluídos 11 estudos, destes 54% recuperados do Pubmed, 91% publicados em inglês. O cuidado bucal mais documentado foi a escovação dentária. Dos achados bucais, gengivite e úlceras foram os eventos mais frequentes.

**Discussão:**

A literatura confirma a presença de achados bucais nesses pacientes relacionados ao seu estado de saúde sistêmico, procedimentos e equipamentos, bem como aos protocolos e cuidados de saúde bucal.

**Conclusões:**

Embora os cuidados bucais sejam prestados em alta proporção em pacientes em estado crítico, é comum a presença de alterações e lesões bucais. A participação do cirurgião-dentista no cuidado integral desses pacientes é fundamental.

## Introduction

 In the global context, the Intensive Care Unit (ICU) is a crucial hospital setting dedicated to managing medium- and high-complexity critically ill patients, ensuring the provision of comprehensive medical care^1^,^2^. However, evidence shows that patients' health often deteriorates after admission to ICUs, as they are highly susceptible to healthcare-associated infections, as well as ventilator-associated pneumonia (VAP), which is among the leading causes of morbidity and mortality^3^.

 Regarding oral health, these patients undergo physiological changes and alterations in oral microbiota due to the use of intraoral attachments and devices, which contribute to states of dysbiosis. Similarly, changes in saliva production, lack of chewing, and other factors increase the risk of gingivitis, periodontal disease, dental caries, and inflammatory or infectious processes in the mucous membranes. Additionally, it is well established that oral care protocols and oral care interventions could be insufficient during patient hospitalization, increasing the risk of developing oral pathologies^4^,^5^ and reducing the patient's quality of life, comfort, and well-being^6^.

 In addition to local disease effects, oral inflammation has been shown to increase the systemic inflammatory burden, directly affecting the health of these patients^7^. Based on this premise, knowing and monitoring the oral health status of critically ill patients could provide timely and valuable information on risk factors and responses to interventions, ultimately improving their hospital stay and future quality of life.

 Numerous tools are available to assess oral health, along with protocols for oral care in the ICU^8^. However, understanding the dynamics of the ICU stay is challenging due to the restrictions of this setting, where patients with special needs stay in accordance with the institutional policies of hospital clinical centers, and safety and control of adverse events are paramount^9^,^10^. In addition, due to the critical conditions of these patients, rigorous institutional protocols must be followed to safeguard their integrity^11^.

 Therefore, the evidence on oral findings in ICU patients remains incipient. A systematic review of the literature is proposed to recognize these previously documented events in comparison with in-hospital patients and support the clinical decision-making processes of the comprehensive healthcare team caring for these patients.

## Materials and Methods

**Study design**

Systematic review (SR) as a type of secondary source research 

**Study protocol**

This systematic review (SR) was conducted following the recommendations of the PRISMA statement and was registered on the PROSPERO platform in June 2023 (CRD42023433917). 

**Eligibility criteria**

The PICO framework used to address the research question and establish the eligibility criteria was as follows: 

P (Population): Soft and hard tissues of the oral cavity in intensive care unit (ICU) patients. 

I (Intervention): ICU hospitalization. 

C (Comparison): In-hospital management. 

O (Outcome): Percentage or frequency of mucositis, herpes, xerostomia, dental caries, gingivitis, and periodontitis. 

Inclusion criteria encompassed original articles published in English or Spanish and available in full-text. Articles that did not document oral findings in ICU patients, case reports, case series, and qualitative studies were excluded. 

**Sources of Information**

An electronic search for articles was conducted in the databases ScienceDirect, Web of Science, PubMed, Oral Dentistry, and Epistemonikos, focusing on publications from 2018 to 2023. 

**Procedure**

The article search in the selected databases was initiated using the search string (((“oral health” OR “dental clinics”) AND (“Intensive Care Units” OR “critical care”) AND (Patients)) tailored for each database (PubMed, Web of Science, Oral Dentist, Science Direct and Epistemonikos), by four of the authors. The articles were obtained by using search filters based on the selection criteria, including publication date and language. Article identification using the respective search string across the five databases was conducted between May 4 and May 18, 2023. 

Subsequently, titles and abstracts from each database were independently reviewed by two peers. In cases of discrepancies between the reviewers, a third team member was consulted to resolve the disagreement by either including or excluding the article from the systematic review. 

The full-text articles in PDF format were downloaded into the reference manager Mendeley and placed in a folder shared with the research team. The titles of the articles were listed and ordered to facilitate the identification of repeated references. After removing duplicates, the remaining articles were exported to the open-access tool Rayyan QCRI^12^, where two researchers reviewed the full texts (https://rayyan.ai/reviews/690450) to ensure they met the selection criteria and documented oral findings in ICU patients. This review process was conducted between June 9 and June 16, 2023. Finally, the methodological quality assessment was conducted using the Joanna Briggs Institute (JBI) assessment tool^13^, which considers aspects such as participant selection criteria, exposure measurement, confounding factors, and outcome measurement, among other aspects, depending on the methodological nature of each study. Data extraction and analysis for each study were recorded in a Microsoft Excel spreadsheet. 

**Analysis**

A descriptive analysis of the data was conducted in Microsoft Excel. The study's data are available for access and consultation on Mendeley Data^14^. 

**Ethical considerations**

Copyright was respected by exercising the right of citation, as stipulated in Law 1915 of 2018 (amending Law 23 of 1982) and Law 1032 of 2006. Additionally, the study adhered to the scientific, technical, and administrative standards for health research outlined in Resolution 008430 of 1993. 

## Results

**Figure 1:**
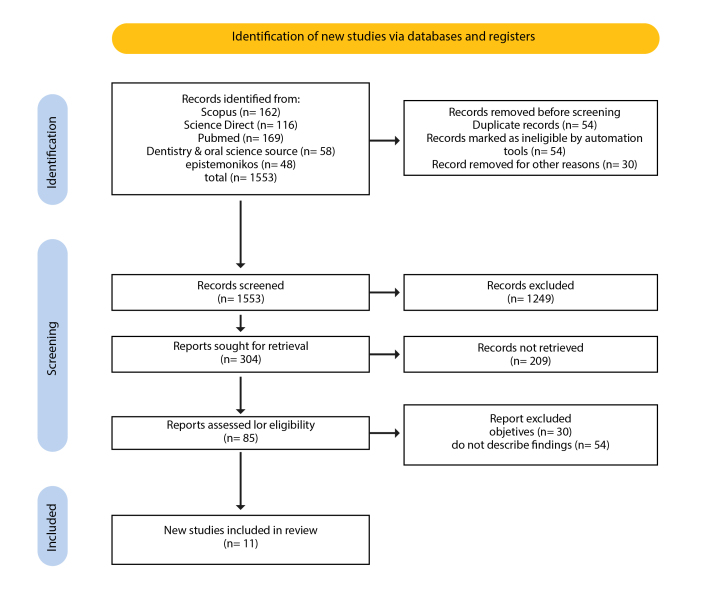
PRISMA flow chart for identification and selection of systematic review articles

A total of 1553 articles were identified. After applying selection filters and conducting a title and abstract review, 95 articles were preselected, and 11 were ultimately included in the systematic review (Figure 1.). 

Regarding the bibliometric characteristics, 54.54% of the articles were retrieved from PubMed, 90.90% were published in English, and 36.36% were published in 2021 period. Brazil was the most frequent country of origin for publications on the subject of interest, accounting for 36.36% (Table 1). 

Regarding oral care provided to the patient, three out of eleven studies (27.27%) did not mention details about oral care. In the remaining eight studies, the most frequently reported care was tooth brushing^15^-^22^ (100% of the evidence) with a daily frequency of three times a day in three studies^19^-^21^, twice a day^22^, every 12 hours in one study^15^, and every 24 hours in two studies^16^,^17^. The use of chlorhexidine mouthwash was reported in eight of the eleven articles^15^,^16^,^18^-^23^ (72.72% of the evidence), although the frequency of its application was not specified. Additionally, two studies describe additional actions such as lip^16^,^22^ and mucosal^22^ hydration, while one study describes specific techniques, such as removal of calculus, scaling and root planing, and atraumatic restorative treatment of caries^19^. 

Regarding the length of stay in the ICU, two studies^24^,^25^ did not report this information, while the other authors recorded durations ranging from one month^15^,^16^ to 39 months^20^. Information on medication intake was mentioned in only 27.27% of the studies. As for comorbidities, 81.81% of the studies reported that participants had at least one comorbidity, including diabetes^16^,^18^,^19^,^23^, hypertension^16^,^19^, and pulmonary compromise^16^,^17^,^19^,^20^,^23^,^24^, among others. Details of these characteristics are described in Table 2. 

In the 11 articles included, a total of 22 oral findings were reported, of which the presence of gingivitis stands out as it was the most frequent event (54.54% of the evidence reviewed) reported in six of the eleven articles^16^,^19^,^20^,^21^,^23^,^24^, followed by ulcers^18^,^20^,^21^,^23^,^25^ (45.45%). Xerostomia, dental caries, and periodontitis were each observed in an equal proportion of studies (36.36%), with these findings being mentioned in four of the 11 articles read^15^-^20^,^23^,^24^. Among other oral findings, edentulism was found in three articles^19^,^20^,^21^, accounting for 27.3%. Findings such as residual roots^16^,^19^, oral abscesses^16^,^23^, angular cheilitis^21^,^23^, and tongue pathologies^20^,^21^ were observed in 18% of the articles. The least frequent findings included lip disorders^18^, oral dysbiosis^24^, dental mobility^20^, and maxillary dental trauma^15^; candidiasis, herpes, aphthous ulcers, and mucositis^23^ were mentioned only once, accounting for 9.09% (Table 2). 

**Table 1:** Bibliometric characteristics of the articles included in the systematic rev

Characteristics	Frequency (n)	Percentage (%)
Database		
PubMed	6	54.54
ScienceDirect	3	27.27
Scopus	2	18.18
Language of publication		
English	10	90.90
Spanish	1	9.09
Type of study		
Observational, cross-sectional, and descriptive	6	54.54
Experimental study or clinical trial	3	27.27
Cohort or longitudinal	2	18.18
Publication year		
2023	3	27.27
2021	4	36.36
2020	2	18.18
2018	2	18.18
Country of publication		
Brazil	4	36.36
Japan	2	18.18
Indonesia	1	9.09
Iran	2	18.18
Korea	1	9.09
Colombia	1	9.09

**Table 2:** Characteristics and results of the studies included in the SR

Author, year	Study design	Comparison group	Tooth brushing frequency	Oral care provided in the ICU	Time spent in ICU	Medication intake	Comorbidities	Place of origin and patient ages	Oral findings identified
Anggraeni et al. 2022^15^	Clinical trial	Oral care with honey vs. traditional care	Every 12 hours	20 ml of chlorhexidine gluconate 0.2%, 20 ml additional honey topically on the oral mucosa, tooth brushing and swabbing techniques	One month	Yes, it is reported; however, the type of medications is not specified.	Autoimmune disease	Indonesia, 18 to 70 years old, n=36 patients, 38.9% men in the control group and 50% men in the intervention group.	Xerostomia, dental maxillary trauma
Steinle et al. 2023^16^	Cohort study	Patients with and without mechanical ventilation	Every 24 hours	Daily oral hygiene with a small toothbrush, vacuum suction, use of chlorhexidine 0.12%, and hydration of the lips with essential fatty acid	One month	Yes, it is reported; however, the type of medications is not specified.	Ventilator-associated pneumonia, diabetes, and arterial hypertension.	Brazil, >18 years old n=207 patients, 51.7% men	Dental caries, gingivitis, periodontal disease, residual tooth roots, and oral abscesses
Satoshi Doi et al. 2021^17^	Analytical cross-sectional study	Perception of dry mouth before and after oral care	Every 24 hours	Brushing teeth with water and cleaning with foam swabs	Twelve months	Not reported	Digestive, respiratory and cardiovascular disease	Japan, >20 and <80 years of age, n=86 patients, 63% men	Xerostomia
Arkia et al. 2023^18^	Analytical cross-sectional study	Oral health status assessment using the BOAS scale for lips, mucous and gums, teeth, saliva, and tongue	Not reported	Tooth brushing, mouth rinse with chlorhexidine	Six months	Not reported	Diabetes	Iran, >18 years old, n=138, 67.4% men	Lip disorder, lip ulcers, xerostomia
Bellissimo et al. 2018^19^	Clinical trial	Dental treatment provided by a dentist vs. routine oral hygiene	Three times a day	Toothbrushing, tongue scraping, removal of calculus, scaling and root planing, atraumatic restorative treatment (ART), rinse with chlorhexidine	24 months	Not reported	Diabetes, hypertension, HIV, cerebral vascular disease, respiratory infections, heart and renal failure, autoimmune disease	Portugal, 17 to 60 years old, n=154 patients, 48% men in the control group and 47.2% men in the intervention group.	Edentulism, caries, gingivitis, residual roots, periodontitis
Takahama et al. 2021^20^	Analytical cross-sectional study	Patients with and without ventilator-associated pneumonia	Three times a day	Daily protocol of oral hygienization of teeth and oral mucosa with toothbrushes and gauze soaked in chlorhexidine digluconate 0.12%.	39 months	Not reported	Cardiovascular disease, trauma, respiratory system diseases, sepsis, gastrointestinal disorders, malignant neoplasms, orthopedic problems, and kidney disease.	Brazil, patients from 18 to 96 years of age, n=663 patients, 62.3% men	Dental loss, presence of removable dental prosthesis, visible cavitation of dental caries, dental mobility, oral/gingival bleeding, coated tongue, hairy tongue, depapillated tongue, and any other mucosal lesions. Ulcers, gingival bleeding.
Dantas Martins et al. 2022^21^	Cohort study	Patients with and without oral alterations	Three times a day	Chlorhexidine 0.2% mouth rinse, tooth brushing	8 months	Not reported	Cancer patient, thrombocytopenia, leukopenia	Brazil, patients over 18 years of age, n=43 patients, 46.5% men	Angular cheilitis, ulcers, edentulism, bleeding, coated tongue, xerostomia.
Ghaempanah et al. 2021^22^	Clinical trial	Intervention group treated with chlorhexidine, toothpaste, and oral moisturizer vs. control group treated with chlorhexidine 0.2% twice a day.	Twice a day	Plaque index, chlorhexidine mouth rinse, toothbrushing, mucosal moisturizer, and lip moisturizing ointment	6 months	Yes, it is reported.	Not reported	Iran, 18 and 65 years old, n=70 patients, 77% male control group and 83% intervention group.	Gingivitis, dental plaque
Sanchez Peña et al. 2020^23^	Analytical cross-sectional study	Patients with and without ventilator-associated pneumonia	Not reported	Chlorhexidine mouth rinse, tooth brushing	5 months	Not reported	Chronic renal failure, heart disease, diabetes mellitus, chronic obstructive pulmonary disease, pulmonary tuberculosis, HIV	Colombia, >18 years old n=99 patients, 58.6% men	Ulcers, gingivitis, periodontitis, candidiasis, herpes, aphthous ulcers, oral abscesses, mucositis, cheilitis.
Yoshino et al. 2023^24^	Descriptive cross-sectional study	Not applicable	Three to eight times a day	Cleaning the oral mucosa and tongue using a sponge brush 3–8 times a day (the frequency was adjusted based on the ventilator status and oral assessment results of the patients). 6 of the 13 patients used mouthwash.	Not reported	Not reported	Ventilator-associated pneumonia, COVID-19, cardiovascular disease, pulmonary disease, neurological disease	Japan, 46 to 60 years old n=13 patients 92% men	Oral dysbiosis, periodontitis, gingivitis, caries.
Kim et al. 2019^25^	Analytical cross-sectional study	Incidence of oral mucosa pressure ulcers	Not reported	Not reported	Not reported	Not reported	Not reported	Korea, >18 years old n=113 patients	Ulcers

**Risk of bias assessment**

The Joanna Briggs Institute (JBI) instruments were used for quality assessment based on the type of study. A visual recognition system using green, yellow, and red colors was used to determine whether the article met (green color), did not have sufficient information (yellow), or did not meet the assessment criteria suggested by the instrument (red color). 

**Figure 2:**
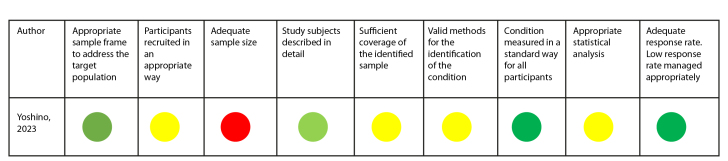
Quality assessment of cross-sectional studies

In the review, the article by Yoshino et al.^24^ was assessed with the tool for cross-sectional studies. According to the questions applied, the study did not provide sufficient information for the assessment of four items and did not provide information regarding the sample size (Figure 2). 

**Figure 3:**
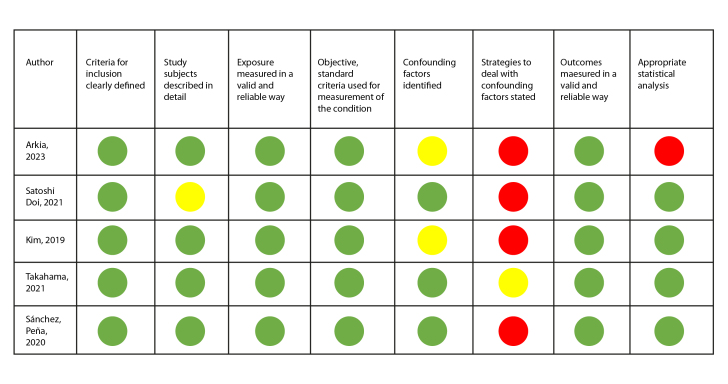
Quality assessment of cross-sectional analytical studies

**Figure 4:**
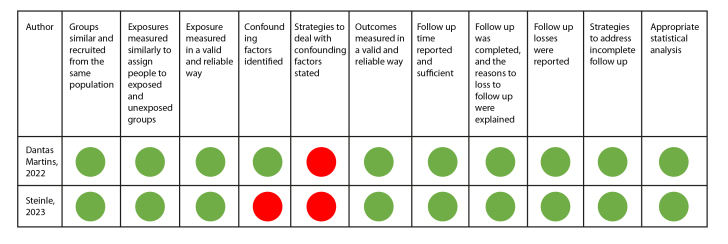
Quality assessment of cohort studies

On the other hand, five of the 11 articles were assessed using the JBI tool for analytical cross-sectional studies, with the most significant limitation being the recognition and control of confounding factors. This limitation was also observed in the assessment of the two cohort studies (Figures 3 and 4). Only the study by Bellisimo et al.^19^ did not document the analysis plan that was executed. 

Overall, the observational studies were of moderate quality and were, therefore, included in the systematic review. In the case of the clinical trials, although their quality assessment was not high, they were maintained in the systematic review due to the valuable information they provided about ICU patients and considering that the identified shortcomings were related to the lack of blinding and randomization of participants in critical care. 

## Discussion

Patients in the ICU are exposed to greater oral health deterioration. Likewise, changes in saliva production, lack of chewing, and poor oral care protocols increase the risk of developing oral pathologies^4^. Accordingly, the aim of this systematic review was to document the oral findings observed in patients who have stayed in the ICU. 

The reviewed literature confirms the occurrence of oral findings in patients during their stay in the ICU^26^. Considering the impact of these events on patient's overall health, the clinical involvement of the dental professional is essential as an integral component of ICU care^27^. 

Regarding the oral findings identified, gingivitis stands out as the most frequently reported event, mentioned in six of the eleven articles^16^,^19^,^20^,^21^,^23^,^24^. According to Trujillo et al. in 2021^28^, gum inflammation in ICU patients is mainly associated with prolonged immobility, dry mouth caused by intubation and mechanical ventilation, and the use of medications that may have side effects impacting the oral cavity. Besides, stress and severe illness can weaken the immune system, leading to dysbiosis in the dental biofilm. 

Following this finding, ulcers were reported in four of the eleven articles included in the SR. According to Trujillo et al.,^28^ the development of ulcers may be related to chronic and degenerative diseases such as diabetes. This condition can increase the risk of oral complications due to its impact on the health of oral tissues by altering their immune response. In addition to patient comorbidities, the process of endotracheal tube fixation during mechanical ventilation in ICU patients has been associated with the formation of continuous pressure ulcers in the oral mucosa^29^,^30^. This finding is consistent with that reported by Chen et al., who documented an incidence of mucosal pressure injuries in ICUs exceeding 80%. The authors highlight as risk factors associated with the development of these injuries the number of medical devices, the model, the duration of use, the number of accessories required based on the patient's condition, and the length of stay in the ICU, among other factors^31^. 

Xerostomia^15^-^17^, dental caries^16^,^18^,^20^,^23^,^24^, and periodontitis^16^,^19^,^23^,^24^ were events reported with significant frequency. These conditions have been associated with limited oral cavity hygiene among critically ill patients. However, evidence indicates that ICU professionals who attend to the patients firsthand often lack the expertise required to provide efficient oral care and may have a limited perception of oral cavity cleanliness. Likewise, Cabrita et al. reported that ICU personnel are unaware of the appropriate equipment for dental plaque removal. Among other variables limiting adequate oral hygiene management in ICU patients include the lack of training, resources, and time dedicated to the oral hygiene of ICU patients^32^. 

In this regard, Kim et al.^33^ mentioned that nurses' knowledge of the oral diseases commonly encountered in ICU patients is insufficient. This highlights the need to provide training and oral care practices to clinical staff working in the ICU. Such training would raise awareness of the importance of documenting oral conditions and reporting the patient's oral health status to provide appropriate treatment. Additionally, it is worth mentioning that the intubation process triggers ventilator-associated pneumonia (VAP), so it would be beneficial to standardize oral care practices in ICU patients^33^. 

Finally, Cañadas-Mota et al.^34^ stress the importance of ICU healthcare personnel being vigilant for early signs of alterations in the patient's oral cavity in order to take basic measures to alleviate dry mouth, such as ensuring adequate hydration and lubrication of the mouth, to improve patient comfort^35^. This requires coordinated work that involves the participation of dentists, who, through their expertise, can support the management of oral hygiene and address the injuries resulting from the care process in the critical care unit^36^. 

The authors highlight the importance of documenting oral findings in ICU patients, recognizing the limitations of conducting longitudinal epidemiological studies given the critical conditions of these patients and the high complexity faced by the medical team. In this sense, the data obtained in this systematic review do not allow for the support of a quantitative meta-analysis. There is a need to join efforts to establish oral examinations as a key to determining the patient's health status and thus making timely interventions. Furthermore, there is a need to establish oral hygiene protocols implemented by clinical institutions. 

## Conclusions

This systematic review showed that the most frequent oral findings in ICU patients were gingivitis, ulcers, dental caries, xerostomia, and periodontitis. These identified findings are attributed not only to pre-existing comorbidities upon ICU admission but also to the specific dynamics of the ICU stay. Most patients undergo mechanical procedures, such as tracheal intubation, which are associated with the appearance of mucosal lesions. Additionally, this condition hinders the oral hygiene process. Therefore, the medical and paramedical teams must have sufficient knowledge to implement oral care protocols. These protocols must involve dentists, who can guide the oral hygiene process and provide critical support to patients during their ICU stay. 
